# Semi-supervised disentangled representation learning for single-cell RNA sequencing data

**DOI:** 10.1093/bib/bbag222

**Published:** 2026-05-11

**Authors:** Haoran Liu, Yuanjie Zou, Zhi Wei

**Affiliations:** Department of Computer Science, New Jersey Institute of Technology, 218 Central Avenue, Newark, NJ 07102, United States; Department of Computer Science, New Jersey Institute of Technology, 218 Central Avenue, Newark, NJ 07102, United States; Department of Computer Science, New Jersey Institute of Technology, 218 Central Avenue, Newark, NJ 07102, United States

**Keywords:** single-cell RNA sequencing, disentangled representation learning, semi-supervised learning, batch effect correction, cell type annotation

## Abstract

Single-cell RNA sequencing (scRNA-seq) data are inherently high-dimensional, and most analysis tools reduce this complexity by projecting the data into a low-dimensional latent space before performing downstream analyses. However, the resulting representations are often entangled, with biological or technical factors such as batch effects and disease stages mixed together, which complicates interpretation. Recent methods have introduced disentanglement mechanisms to improve interpretability, but they typically require large amounts of well-annotated data to perform well or are limited to factors with only a few categories. To address these challenges, we propose **SCDRL** (Semi-Supervised Disentangled Representation Learning for Single-Cell RNA Sequencing Data), a method that uses gene expression profiles together with a small proportion of labeled samples to learn disentangled representations that separate batch effects, cell types, and other biological signals, thereby enhancing interpretability. Unlike existing approaches, SCDRL generalizes from factors with only a few categories to complex settings involving more than 10 cell types. Experiments on both simulated and real-world datasets demonstrate that SCDRL consistently outperforms existing disentangled representation learning methods for scRNA-seq data, even when only 5% of labeled samples are available.

## Introduction

Single-cell RNA sequencing (scRNA-seq) has revolutionized the study of cellular heterogeneity by enabling the profiling of gene expression at single-cell resolution. Unlike bulk RNA sequencing, which measures average gene expression across large populations of cells and can obscure rare or transient states, scRNA-seq captures subtle differences between individual cells. This capability has led to breakthroughs in understanding complex biological systems, uncovering previously unknown cell types, reconstructing developmental trajectories, and providing insights into disease mechanisms across fields such as immunology, oncology, and developmental biology [1–4].

Despite these advantages, scRNA-seq data present significant computational challenges. Each cell is represented by thousands of gene expression measurements, resulting in high dimensionality. The data are also sparse and noisy, often due to technical dropout events where transcripts present in a cell fail to be captured during sequencing [5]. Furthermore, experiments are commonly conducted across multiple batches, each introducing technical artifacts through differences in reagents, instrumentation, or sequencing depth. These batch effects add systematic, non-biological variation that can obscure biological signals and hinder integration across datasets [6]. In addition, genuine biological variation, such as donor heterogeneity, environmental influences, and temporal dynamics, further complicates analysis.

To address these challenges, computational biologists often employ representation learning techniques that transform raw, high-dimensional profiles into lower-dimensional latent spaces while preserving biological structure and reducing noise. Traditional methods such as principal component analysis (PCA), t-distributed stochastic neighbor embedding, and Uniform Manifold Approximation and Projection (UMAP) have been widely applied for visualization and clustering [7, 8]. More recently, deep learning models, particularly variational autoencoders (VAEs), have gained traction for learning flexible and expressive latent representations that simultaneously capture biological signals and technical artifacts [9–12].

A major challenge is the entanglement of latent factors. Most representation learning methods produce mixed representations that fail to separate biological sources of variation (e.g. disease state or cell type) from technical artifacts (e.g. batch effects). Even methods designed to remove nuisance factors may inadvertently eliminate relevant biological variation [13, 14], potentially obscuring rare cell populations and compromising downstream analyses such as trajectory inference and differential expression [15].

Disentangled representation learning has therefore emerged as a promising paradigm. The goal is to factorize the latent space into interpretable components, each corresponding to a specific underlying source of variation. In the context of scRNA-seq, this involves learning latent dimensions that capture biological and technical attributes such as batch, disease state, or cell type, while isolating residual variation into a separate component. Disentangled representations enable more robust downstream analyses, including virtual perturbation, cross-condition generalization, and mechanistic interpretation of cellular programs [16, 17].

Another key limitation of many deep representation learning methods is their reliance on extensive supervision. Supervised models assume that sufficient labels, such as cell types or experimental conditions, are available to guide learning. However, in practice, annotated data are scarce. Labeling scRNA-seq data requires labor-intensive expert curation, often based on marker gene expression and reference to existing knowledge bases. In many exploratory studies, only a small fraction of cells are annotated, and even when labels exist, they may be noisy, incomplete, or inconsistent across studies [18, 19].

Several approaches have been developed to address these challenges, though each has its limitations. **Seurat** [20], a widely used toolkit, integrates datasets using canonical correlation analysis and mutual nearest neighbors to align batches and identify shared cell states. **scVI** [10] employs unsupervised variational inference to model gene expression and correct for batch effects. Although Seurat and scVI are widely adopted, they are not explicitly designed for disentanglement. Other methods, such as **scDisInFact** [21], explicitly disentangle shared and condition-specific signals using a supervised multi-encoder framework, but they require batch information for every cell as input. Similarly, **scDisco** [22] assumes that batch information is available for all cells, which restricts its use in practical settings where annotations are sparse. Factorization-based approaches such as **scINSIGHT** [23] use non-negative matrix factorization to isolate batch effects or other factors, but cell type information often remains entangled, requiring additional clustering of the learned representations.

More closely related to our work, **biolord** [24] proposes a variational generative model that disentangles known and unknown factors of variation and enables virtual perturbation prediction. In settings with limited supervision, a biolord may struggle to generalize, fail to capture rare cell types, or collapse to trivial solutions.

In this study, we introduce **SCDRL** (*Semi-Supervised Disentangled Representation Learning for Single-Cell RNA Sequencing Data*), a novel deep generative framework designed for robust and interpretable analysis of scRNA-seq data with minimal labeled supervision. Built on a modular VAE architecture, SCDRL partitions the latent space into multiple factors of interest, each represented by a dedicated encoder. These include categorical biological attributes such as cell type or disease condition, as well as a residual factor capturing unexplained variation. By combining semi-supervised classification, entropy regularization, residual regularization constraints, and reconstruction penalties, SCDRL encourages each latent dimension to specialize in modeling a distinct factor.

SCDRL is conceptually related to the factor-aware disentanglement framework introduced in ZeroDIM [25]. To clarify the conceptual design of SCDRL and its relationship to prior disentangled representation learning frameworks, we briefly summarize its architectural intuition below.

Compared with ZeroDIM, which was developed for low-dimensional vision datasets with dense inputs and limited factor complexity, SCDRL incorporates several extensions required for realistic single-cell RNA-seq analysis. In particular, SCDRL is designed to address the unique statistical and biological challenges of scRNA-seq data, including extreme sparsity, high dimensionality, complex multi-class biological factors, and severe label scarcity.

To this end, SCDRL incorporates explicit batch-effect modeling, dedicated factor-specific encoders for biological conditions and multi-class cell types, and a residual regularization mechanism tailored to sparse and noisy gene expression data. In addition, SCDRL adopts reconstruction and optimization strategies compatible with high-dimensional count-based gene expression distributions, enabling stable training under limited supervision.

**Figure 1:**
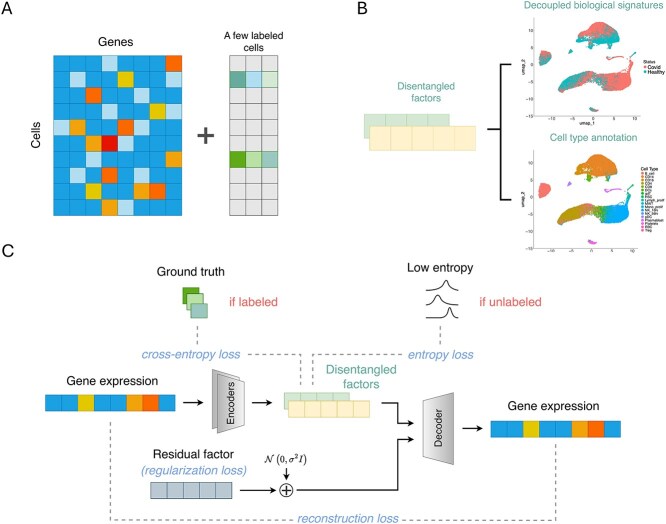
SCDRL Architecture Overview. (A) Input to the model consists of gene expression data along with a small fraction of labeled cells. In our setting, a labeled cell is defined as one for which all factor labels are available. (B) The model outputs disentangled factors with biological interpretations. (C) Model framework: for each factor, a dedicated encoder is responsible for its extraction. The latent space is partitioned into interpretable factors and a residual component, both of which are used to reconstruct the original gene expression vector. Specifically, if a cell is labeled, we compute the cross-entropy loss; if it is unlabeled, we apply an entropy regularization term.

Building on the VAE paradigm, SCDRL partitions the latent space into multiple interpretable components corresponding to distinct biological and technical factors, such as batch, treatment condition, and cell type, while reserving a residual component to capture unmodeled variation. This modular design encourages specialization of factor-specific latent dimensions and facilitates biologically grounded disentanglement. Figure 1 provides a conceptual overview of the architecture.

Moreover, SCDRL is designed to perform effectively even when only a small fraction of cells (e.g. 5%) are labeled. It leverages entropy-based regularization on unlabeled cells to enforce confident predictions and applies residual regularization to isolate noise and unmodeled variation.

We evaluate SCDRL across diverse scenarios, including synthetic datasets with known ground-truth factors and real-world datasets representing multiple biological contexts. Comparisons with existing methods demonstrate that SCDRL consistently achieves superior performance in recovering true cell types, correcting batch effects, and disentangling complex condition-specific signals, even under stringent label constraints.

In summary, SCDRL provides a flexible, scalable, and interpretable framework for representation learning in scRNA-seq analysis. It bridges the gap between fully supervised and unsupervised models, offering a practical solution for real-world applications where annotations are sparse, data are noisy, and biological variation is multifactorial.

## Materials and methods

In this section, we present the technical framework of **SCDRL**, a deep generative model designed for analyzing scRNA-seq data under limited supervision. The main objective of SCDRL is to learn a structured, interpretable low-dimensional representation of each cell’s gene expression profile. This representation aims to disentangle known biological and technical factors, such as cell type, treatment condition, or batch, from unobserved or residual variation. Unlike fully supervised methods, SCDRL is designed to perform well even when only a small subset of cells is labeled.

### Mathematical framework

Let $X = [x_{1}, x_{2}, \dots , x_{n}]$ be a matrix of gene expression profiles where each $x_{i} \in \mathbb{R}^{d}$ denotes the high-dimensional vector of expression levels for cell $i$. We assume that $x_{i}$ is generated by a function $G$ acting on $k$ interpretable latent factors $\{f_{i}^{1}, f_{i}^{2}, \dots , f_{i}^{k}\}$ and a residual latent variable $r_{i}$:


1 \begin{eqnarray*}& x_{i} = G(f_{i}^{1}, f_{i}^{2}, \dots, f_{i}^{k}, r_{i}).\end{eqnarray*}

Each $f_{i}^{j}$ represents a categorical variable such as cell type or batch. The residual $r_{i}$ models unstructured variation i.e. not captured by predefined factors, including technical noise or unidentified biological signals.

### Factor-specific encoders and semi-supervised learning

For each factor $j$, we define a separate encoder $E^{j}$ that maps input $x_{i}$ to a distribution over the categorical values of that factor. When supervision is available, i.e. the label for $f_{i}^{j}$ is known, we apply cross-entropy loss:


2 \begin{eqnarray*}& \mathcal{L}_{\mathrm{cls}}=\sum_{i=1}^{n} \sum_{j=1}^{k} \ell(i, j) \cdot H_{ce}\left(\operatorname{Softmax}\left(E^{j}\left(x_{i}\right)\right), f_{i}^{j}\right),\end{eqnarray*}

where $H_{ce}(\cdot , \cdot )$ is the cross-entropy function and $\ell (i, j)$ is an indicator of label availability:


3 \begin{eqnarray*}& \ell(i, j) = \begin{cases} 1 & \text{if label for factor } j \text{ is available for cell } i, \\ 0 & \mathrm{otherwise.} \end{cases}\end{eqnarray*}

To encourage confident predictions for unlabeled cells, we apply entropy regularization [26]:


4 \begin{eqnarray*}& \mathcal{L}_{\mathrm{ent}} = \sum_{i=1}^{n} \sum_{j=1}^{k} (1 - \ell(i, j)) \cdot H_{ent}(\mathrm{Softmax}(E^{j}(x_{i}))).\end{eqnarray*}

This term drives the model to produce sharp, low-entropy predictions for unlabeled factors, mimicking one-hot distributions and reinforcing semantic consistency.

### Residual regularization and latent composition

To ensure that factor encoders capture their intended variation and that the residual vector $r_{i}$ does not absorb informative signal, we impose two forms of regularization:

**1. Noise injection:** Gaussian noise is added to the residual to reduce overfitting:


5 \begin{eqnarray*}& r_{i}^{\prime} = r_{i} + \mu, \quad \mu \sim \mathcal{N}(0, I).\end{eqnarray*}

**2. Magnitude penalty:** A squared $\ell _{2}$ norm is applied to keep the residual compact:


6 \begin{eqnarray*}& \mathcal{L}_{\mathrm{res}}=\sum_{i=1}^{n}\left\|r_{i}\right\|^{2}.\end{eqnarray*}

Together, these constraints guide the residual to encode only truly unmodeled variation and ensure that interpretable factors are not underutilized.

### Reconstruction and full loss function

To reconstruct each cell’s gene expression $x_{i}$, the decoder $G$ takes the inferred latent representation. For each factor $f^{j}$, we use the true label if known, or the softmax prediction otherwise:


7 \begin{eqnarray*}& \tilde{f}_{i}^{j} = \begin{cases} f_{i}^{j} & \text{if } \ell(i, j) = 1, \\ \mathrm{Softmax}(E^{j}(x_{i})) & \mathrm{otherwise.} \end{cases}\end{eqnarray*}

The decoder reconstructs $x_{i}$ using $\tilde{f}_{i}^{1}, \dots , \tilde{f}_{i}^{k}$ and $r_{i}^{\prime}$:


8 \begin{eqnarray*}& \mathcal{L}_{\mathrm{rec}} = \sum_{i=1}^{n} \phi(G(\tilde{f}_{i}^{1}, \dots, \tilde{f}_{i}^{k}, r_{i}^{\prime}), x_{i}),\end{eqnarray*}

where $\phi (\cdot , \cdot )$ denotes a reconstruction loss, which typically mean squared error for normalized data, or a distributional loss such as negative binomial for raw count data [27].

The full loss function integrates all objectives:


9 \begin{eqnarray*}& \mathcal{L}_{\mathrm{total}} = \mathcal{L}_{\mathrm{cls}} + \lambda_{1} \mathcal{L}_{\mathrm{ent}} + \lambda_{2} \mathcal{L}_{\mathrm{res}} + \lambda_{3} \mathcal{L}_{\mathrm{rec}},\end{eqnarray*}

where $\lambda _{1}$, $\lambda _{2}$, and $\lambda _{3}$ are scalar hyperparameters controlling the relative importance of each component.

### Implementation details

SCDRL is implemented in PyTorch [28] and supports flexible configuration of factor encoders, latent dimensions, and regularization weights. Source code and documentation are available at https://github.com/Haoran-Liu/SCDRL, which also includes detailed instructions for building the conda environment required to run the code.

## Results

We evaluate the performance of **SCDRL** on both synthetic and real-world scRNA-seq datasets. These include a simulation study with controlled ground truth, a cross-species mouse–human dataset, and a COVID-19 dataset. Across all datasets, we compare the performance of SCDRL against three competing methods: **biolordscVI**, and **Seurat**. All hyperparameters are fixed across datasets to ensure fairness and generality.

We primarily evaluate all methods based on their ability to recover true cell type labels, in addition to their capacity to disentangle biological factors. In practice, cell type clustering is often the primary focus, and scVI and Seurat are not explicitly designed to disentangle multiple biological signals. Our experiments are designed to reflect realistic challenges in scRNA-seq analysis, including sparse annotations, technical noise, and dataset heterogeneity. For evaluation, we use UMAP visualizations along with F1 score and Adjusted Rand Index (ARI) as quantitative metrics.

For semi-supervised methods such as biolord, predictions can be directly output by the model for each attribute of unlabeled cells; thus, we report F1 scores to measure classification accuracy. In contrast, scVI, a deep generative model for single-cell RNA sequencing data, and Seurat only produce clustering results; therefore, we assess their performance using ARI to compare clustering quality against the ground truth.

The cell coordinates in the UMAP plots were generated by Seurat for a unified visualization, and the cell annotations correspond to the results of the respective methods.

### Competing methods

Before presenting dataset-specific results, we briefly describe the competing methods considered in this study. While other disentangled representation learning methods, such as **scDisInFact** [21] exist, they were not included for direct comparison. For instance, scDisInFact does not explicitly disentangle cell type information. Instead, it disentangles biological factors such as batch and disease stage from the learned cell representations, within which cell type information resides. Consequently, additional clustering steps are required to infer cell types. This makes direct comparison less appropriate with semi-supervised classification frameworks such as SCDRL and biolord, which can simultaneously disentangle cell type information.

**biolord** [24] is a disentangled representation learning framework for single-cell RNA-seq data. It uses a semi-supervised VAE to separate biological and technical sources of variation. Partial annotations are incorporated to guide latent space disentanglement, enabling interpretable representations when labels are limited. Biolord employs condition-specific priors and regularization strategies to encourage separation of attributes such as cell type, condition, and batch.

**scVI** [10] is an unsupervised VAE framework for scRNA-seq data. It performs dimensionality reduction and batch correction by modeling raw expression counts with a negative binomial likelihood and incorporating batch as a covariate. While powerful for integration and denoising, scVI does not explicitly disentangle biological factors and requires post hoc interpretation of its latent space.

**Seurat** [20] is a widely used R toolkit for single-cell RNA-seq analysis. It performs batch correction and clustering through canonical correlation analysis and mutual nearest neighbors, and more recently supports multimodal integration. Seurat is highly effective for exploratory analysis but does not learn generative latent representations or explicitly disentangle factor-specific variation.

For semi-supervised methods (SCDRL and biolord), we repeated each experiment 10 times with different random seeds to select varying sets of labeled cells, thereby reducing bias from random label assignment. For scVI and Seurat, the resolution parameter that controls the number of clusters generated was carefully tuned to match the true number of cell types, ensuring a fair comparison when computing the ARI.

### Simulation dataset

**Figure 2:**
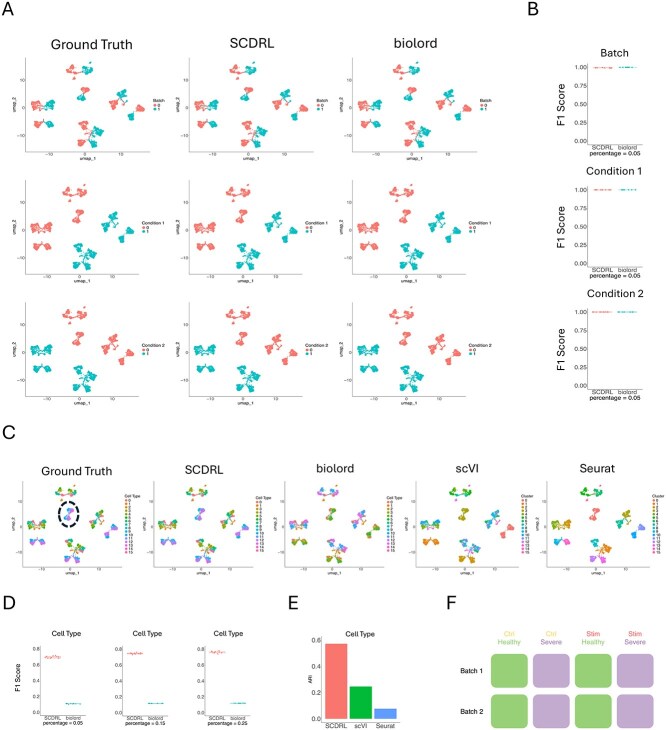
Simulation Dataset. (A) Visualization of annotations from SCDRL and biolord compared with the ground truth. The first row represents batch labels, the second corresponds to condition 1, and the third to condition 2. (B) F1 scores for batch, condition 1, and condition 2 classification results. (C) UMAP plots showing that SCDRL closely matches the ground truth cell type annotations. (D) Performance of SCDRL and biolord in terms of cell type classification when provided with different proportions of labeled cells. (E) ARI results in terms of cell type, where SCDRL uses 5% labeled data. (F) The complete simulation dataset consists of 8 subsets with different factor settings.

We first evaluated SCDRL using a simulation study to assess its ability to disentangle latent factors under controlled conditions. A synthetic scRNA-seq dataset was generated with SymSim [29], following the setup of scDisInFact [21]. The dataset includes two batches, two condition 1 types (Ctrl and Stim), two condition 2 types (Healthy and Severe), and 16 cell types (labeled 0–15). Each combination of batch and conditions produces a unique subset, resulting in eight subsets in total (Fig. 2F). The dataset contains approximately 10 000 cells and 500 genes. All cell types contained 660 cells, except for cell type 2, which was defined as a rare population with only 200 cells.

Classification of batch, condition 1, and condition 2 is relatively straightforward since each has only two categories. As shown in Fig. 2A and B, both SCDRL and biolord achieved strong classification performance under the semi-supervised setting with only 5% labeled cells.

In contrast, cell type classification is more challenging due to the presence of 16 categories. Figure 2C illustrates the results under 5% supervision. SCDRL produced embeddings and predictions that closely matched the ground truth, while biolord performed well for binary factors but struggled with the multi-class cell type task, frequently misassigning labels. The clustering outputs of scVI and Seurat were also less accurate. For example, the circled two populations in Fig. 2C were correctly distinguished only by SCDRL, whereas biolord incorrectly predicted their labels, scVI and Seurat erroneously merged them into a single cluster.

Figure 2D reports cell type classification performance as the proportion of labeled cells increases from 5% to 25%. SCDRL shows clear performance gains with additional labels, while biolord exhibits only minor improvements.

ARI comparisons are shown in Fig. 2E. With just 5% labeled cells, SCDRL significantly outperformed scVI and Seurat. To reduce sampling bias, the experiment was repeated 10 times with different random subsets of labeled cells, and the results were averaged. Among the clustering methods, scVI performed better than Seurat, likely because scVI was provided with batch information for each cell, enabling more effective batch removal.

These results demonstrate SCDRL’s advantage in classification, scaling from binary attribute prediction to multi-class cell type classification. As shown in Fig. 2A and C, the UMAP embeddings annotated by SCDRL yield disentangled representations that align closely with the ground truth across batches, conditions, and cell types.

### Mouse–human cross-species dataset

We next assessed SCDRL on a mouse–human cross-species dataset, where batch effects are particularly severe due to species divergence. This dataset was curated by scvi-tools [14] and includes orthologous cell populations across species. It contains 10 000 cells and 1768 genes. The central challenge is to recover shared cell types while removing species-specific technical biases.

**Figure 3:**
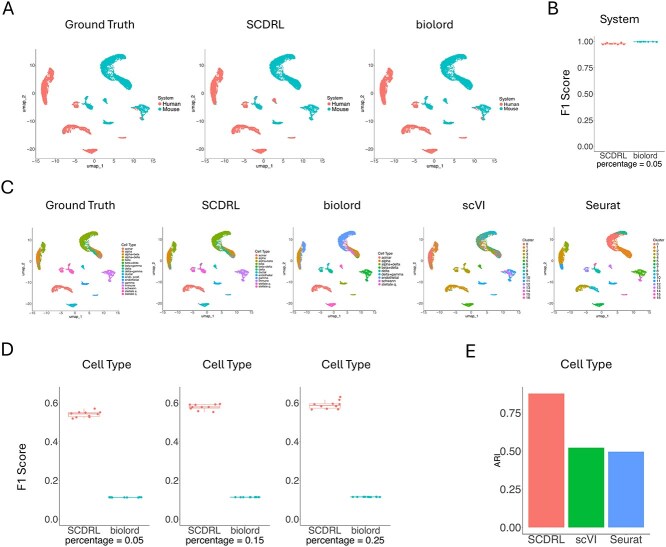
Mouse–Human Cross-Species Dataset. (A) Visualization of system annotations (human or mouse) from SCDRL and biolord compared with the ground truth. (B) F1 scores of system classification results. (C) UMAP plots of cell types across different methods. (D) Performance of SCDRL and biolord in terms of cell type classification when provided with different proportions of labeled cells. (E) ARI results in terms of cell type, where SCDRL uses 5% labeled data.

Figure 3A shows that system-level classification (human vs. mouse) is relatively straightforward, as it represents a binary classification task. Both SCDRL and biolord achieved high F1 scores with only 5% of labeled cells (Fig. 3B).

Cell type classification, however, is considerably more challenging because of the larger number of categories and the presence of closely related subtypes. As shown in Fig. 3C, the UMAP embeddings annotated by SCDRL closely matched the ground truth. In contrast, biolord frequently misclassified cell types, particularly when distinguishing between similar subtypes. We also found that although both SCDRL and biolord failed to predict certain minor populations, the overall annotations from SCDRL aligned much more closely with the ground truth. Specifically, SCDRL successfully annotated 12 out of 17 cell types, compared with only 9 out of 17 for biolord. scVI and Seurat performed even worse, often merging distinct subpopulations into single clusters. As stated earlier, the resolution parameters of scVI and Seurat were carefully tuned to output the same number of clusters as the true number of cell types, ensuring a fair comparison when computing the ARI.

Figure 3D reports classification performance across different proportions of labeled cells. As in the simulation study, SCDRL showed clear improvements as more labeled data were provided, while biolord’s gains were minimal.

Finally, ARI comparisons (Fig. 3E) demonstrate that with only 5% labeled cells, SCDRL outperformed both scVI and Seurat. The advantage of SCDRL reflects its ability to disentangle system-level and cell-type–specific variation simultaneously, thereby achieving accurate cross-species classification under semi-supervised conditions.

### COVID-19 dataset

We next evaluated SCDRL on a subset of the COVID-19 study by Stephenson *et al.* [30], processed and provided by scvi-tools [31]. This dataset consists of 30 000 cells and 16 743 genes across two batches and 18 annotated cell types.

**Figure 4:**
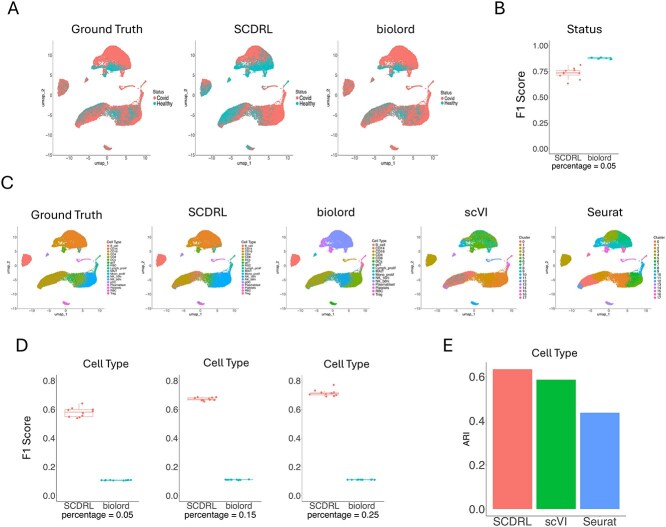
COVID-19 Dataset. (A) Visualization of status annotations (COVID-19 or Healthy). from SCDRL and biolord compared with the ground truth. (B) F1 scores of status classification. (C) UMAP plots of cell types across different methods. (D) Performance of SCDRL and biolord in terms of cell type classification when provided with different proportions of labeled cells. (E) ARI results in terms of cell type, where SCDRL uses 5% labeled data.

Although SCDRL was slightly outperformed by biolord in disease state classification (Fig. 4A and B), SCDRL demonstrated stronger performance in multi-class cell type classification. As shown in Fig. 4C, the UMAP embeddings annotated by SCDRL closely matched the ground truth. In contrast, biolord often failed to resolve rare cell types and misclassified closely related populations. scVI and Seurat also struggled, frequently merging distinct populations into single clusters.

Fig. 4D reports the effect of varying the proportion of labeled cells. Consistent with previous datasets, SCDRL showed clear improvements as more labeled data were provided, whereas biolord exhibited only minor gains.

Finally, ARI results in Fig. 4E show that with only 5% labeled cells, SCDRL outperformed both scVI and Seurat. Together, these results highlight SCDRL’s robustness in handling large, heterogeneous datasets with complex batch effects and disease-related variation.

#### Disentanglement performance

To evaluate the disentanglement performance of each method, we compute a suite of widely used disentanglement metrics, including Mutual Information Gap (MIG) [32], Attribute Predictability Score (SAP) [33], Disentanglement, Completeness, and Informativeness (DCI) [34], Hungarian matching [35], and Spearman correlation. For a fair comparison, the latent dimensionality of scVI and Seurat is matched to that of SCDRL and biolord; for Seurat, latent dimensions correspond to principal components obtained via PCA. All metrics are computed between the learned latent dimensions used for downstream tasks and the known ground-truth factors.

**Figure 5:**
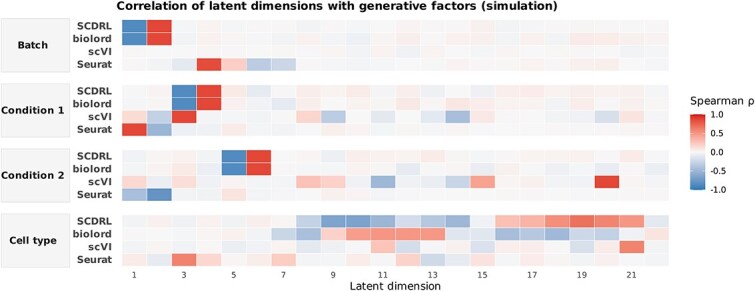
Spearman of latent dimensions with generative factors (simulation dataset) Heatmaps of Spearman’s $\rho$ between each latent dimension and the four ground-truth generative factors (Batch, Condition 1, Condition 2, and Cell type). For each factor, rows correspond to methods (Seurat, scVI, biolord, and SCDRL) and columns to latent dimensions; the intensity of the shading corresponds to correlation strength ($\rho \in [-1,1]$). Disentanglement methods (SCDRL and biolord) show sparse, factor-aligned high correlations, whereas scVI and Seurat exhibit weaker or more diffuse associations.

For well-disentangled representations, latent dimensions associated with a given factor should exhibit strong correlations (close to $\pm$1) with that factor while remaining weakly correlated with unrelated factors. As shown in Fig. 5, disentanglement-based methods such as SCDRL and biolord display clear block-structured correlation patterns in the simulation dataset. For example, only dimensions 1–2 of SCDRL show strong correlations with the batch factor, while the remaining dimensions exhibit minimal association, indicating successful factor separation. In contrast, scVI and Seurat are not explicitly designed to disentangle latent factors, and their latent dimensions exhibit weak, diffuse, or inconsistent correlations across multiple factors, reflecting entangled representations. Notably, Seurat mixes information from multiple conditions into its leading principal components, further highlighting its lack of disentanglement.

For the cell type factor, SCDRL and biolord correctly show weak correlations in dimensions 1–6, dimensions not intended to encode cell type information, while stronger correlations are concentrated in the remaining dimensions. In contrast, scVI and Seurat do not exhibit clear separation, with individual dimensions correlating inconsistently with multiple factors.

**Figure 6:**
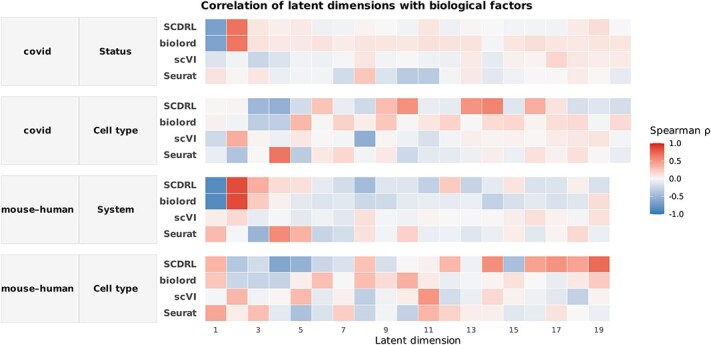
Spearman correlations of latent dimensions with biological factors (real datasets). Spearman’s $\rho$ between latent dimensions and annotated biological factors in two real datasets (COVID: Status and Cell type; mouse–human: System and Cell type). Rows denote methods (Seurat, scVI, biolord, and SCDRL) and columns denote latent dimensions; the intensity of the shading corresponds to correlation strength. ($\rho \in [-1,1]$). Despite increased noise and complexity in real data, SCDRL and biolord retain clearer and more consistent factor-specific correlation patterns than non-disentanglement baselines.

Figure 6 presents Spearman correlation analyses on two real datasets. Consistent with the simulation results, dimensions 1–2 correspond primarily to the system factor in the mouse–human dataset or to disease status in the COVID-19 dataset, while the remaining dimensions are associated with cell type. Although correlations are generally weaker in real datasets due to increased biological complexity and technical noise, SCDRL and biolord still recover the expected correlation structure on the COVID-19 dataset. In the more challenging mouse–human setting, neither method shows strong correlations between cell type and latent dimensions, reflecting the difficulty of cross-species cell type alignment; however, both methods reliably capture strong correlations between system identity (mouse vs. human) and dimensions 1–2, demonstrating successful disentanglement of the dominant biological factor.

**Figure 7:**
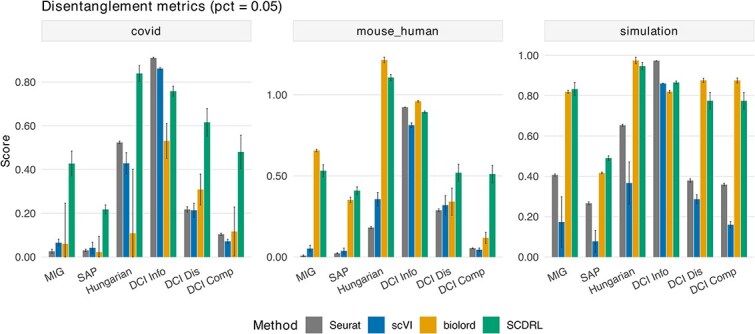
Disentanglement performance across metrics and datasets with 5% labeled data. Grouped bar plots for three datasets (COVID, mouse–human, and simulation). The x-axis lists disentanglement metrics (MIG, SAP, Hungarian alignment, and DCI Info/Dis/Comp), and the y-axis shows the mean score across random seeds; error bars indicate $\pm$1 standard deviation. Each bar represents a different method (Seurat, scVI, biolord, and SCDRL).

We further quantify disentanglement using predictor-based and information-based metrics [36] to provide a systematic comparison. Figure 7 reports quantitative disentanglement scores (MIG, SAP, Hungarian matching, and DCI metrics) across the COVID-19, mouse–human, and simulated datasets under a semi-supervised setting with only 5% labeled cells. Across all datasets, SCDRL consistently achieves comparable or superior disentanglement scores with reduced variance compared to Seurat, scVI, and biolord. In particular, SCDRL achieves high MIG and SAP scores, indicating improved alignment between latent dimensions and ground-truth factors, while maintaining high DCI Informativeness, reflecting better predictive capacity of individual latent components. On the more challenging mouse–human and COVID-19 datasets, SCDRL preserves robust disentanglement performance, whereas competing methods exhibit pronounced degradation, especially in DCI Disentanglement and Completeness. In the simulation benchmark, SCDRL approaches near-oracle performance on Hungarian matching and DCI metrics, demonstrating its ability to recover factor-specific latent structure when ground truth is available. Together, these results show that SCDRL learns more factorized and interpretable representations than existing methods, even under severe label scarcity and across diverse settings.

## Discussion

We introduced **SCDRL**, a semi-supervised deep generative framework that learns disentangled, factor-specific representations for scRNA-seq data under sparse annotation. Across a controlled simulation, a mouse–human cross-species dataset, and a large COVID-19 cohort, SCDRL consistently produced accurate and interpretable latent structures, outperforming widely used baselines in multi-class cell type recovery while remaining competitive on binary attributes such as disease status. These results demonstrate that combining modular disentanglement with semi-supervised objectives is an effective strategy for addressing the high dimensionality, sparsity, and heterogeneity characteristic of scRNA-seq data [5, 6, 9, 11].

A central contribution of SCDRL lies in its modular encoder design, which allocates distinct latent components to biological and technical factors (e.g. cell type, condition, batch) while relegating unexplained signal to a residual component. This factorization improved interpretability and downstream performance. In the simulation benchmark, SCDRL maintained strong accuracy on binary factors (batch and two conditions) and, crucially, generalized to the substantially harder multi-class cell type task with 16 categories. On real datasets, SCDRL preserved cell type structure even across severe batch or species shifts, where scVI and Seurat often merged distinct populations and biolord misassigned labels. These observations align with the broader promise of disentangled representations for making latent spaces more semantically meaningful and robust to confounders [10, 16, 21, 24].

Another advantage of SCDRL is its sample efficiency under sparse supervision. The model retained high performance with only 5% labeled cells, a regime that reflects practical constraints in single-cell studies where expert annotation is costly and uneven [18, 19]. Entropy-based regularization on unlabeled cells encouraged confident, low-entropy predictions [26], while residual regularization reduced leakage of structured signal into the residual latent variable. Together, these design choices allowed SCDRL to learn meaningful factorization without extensive labels. In contrast, methods that either do not natively use labels (e.g. Seurat, scVI) or require extensive supervision and factor metadata (e.g. scDisInFact) can be disadvantaged in label-scarce scenarios [10, 20, 21].

The cross-species experiment further emphasized SCDRL’s robustness to biologically grounded batch-like differences. SCDRL correctly separated species identity (mouse vs. human) while recovering orthologous cell types more faithfully than comparison methods. In the COVID-19 cohort, SCDRL was slightly outperformed by biolord in binary disease-state classification but surpassed all baselines in multi-class cell type recovery and clustering quality, particularly for rare or closely related populations. This pattern suggests that SCDRL’s advantage scales with factor complexity, consistent with its design goal of accurate multi-class disentanglement.

In practical single-cell workflows, latent representations are expected to support multiple analytical goals, including batch correction, clustering, annotation transfer, and condition-aware analysis [11, 13]. SCDRL’s modularity enables a unified framework that can perform all these tasks simultaneously: integrating batches, classifying labeled factors, and discovering unlabeled structures. Practically, we recommend defining a small set of factors of interest (e.g. batch, condition, cell type), providing sparse but high-confidence labels when available, and applying moderate residual regularization to prevent factor leakage. These principles align with best practices for robust representation learning and data integration in scRNA-seq analysis [9, 14].

Despite its advantages, SCDRL also has limitations. Theoretical identifiability of disentanglement is not guaranteed without strong inductive biases [16]. Although our architecture and regularization improved practical separation, perfect interpretability cannot be ensured. Moreover, model performance depends on correctly specifying the factors of interest; missing or mis-specified covariates may cause a structured signal to leak into the residual component. While we demonstrated computational efficiency for datasets of up to tens of thousands of cells, scaling to atlas-level data with millions of cells will require distributed or mixed-precision training optimized for sparse matrices [27, 37]. Additionally, SCDRL currently focuses on transcriptomic measurements, and future work should explore extensions to multimodal assays such as scATAC-seq, CITE-seq, and spatial transcriptomics [20, 38, 39].

Two directions emerge for future development. First, incorporating causal or graph-based priors could enhance stability and interpretability, especially for interacting factors such as condition-by-cell-type effects [40]. Second, integrating perturbation-aware generative modeling would enable counterfactual predictions across conditions or donors, complementing recent advances in single-cell perturbation modeling [17]. Further work on automated factor selection, active label acquisition [41], and uncertainty quantification could improve SCDRL’s usability in exploratory and large-scale analyses.

**Key Points**

- We propose SCDRL (Semi-Supervised Disentangled Representation Learning for Single-Cell RNA Sequencing Data), a deep generative framework that learns disentangled representations of scRNA-seq data from gene expression profiles together with only a small proportion (as low as 5%) of labeled cells.
- SCDRL uses a modular VAE with factor-specific encoders and a residual component, jointly optimized with cross-entropy, entropy regularization, residual regularization, and reconstruction losses to separate batch effects, cell types, and other biological signals into interpretable factors.
- Unlike prior disentangled methods limited to factors with only a few categories, SCDRL generalizes to realistic scRNA-seq settings involving more than 10 cell types, high dimensionality, and severe label scarcity.
- Experiments on simulated, mouse–human cross-species, and COVID-19 datasets show that SCDRL consistently outperforms biolord, scVI, and Seurat in cell type recovery, batch correction, and condition-specific signal disentanglement, even with only 5% labeled cells.

## Data Availability

The datasets analyzed in this study are publicly available from the original sources listed below. **Simulation dataset:** Available at our project repository on GitHub (link provided below). **Mouse–Human Cross-Species Dataset:** Accessible via the scvi-tools tutorial at https://docs.scvi-tools.org/en/stable/tutorials/notebooks/scrna/sysVI.html. **COVID-19 Dataset:** Accessible via the scvi-tools tutorial at https://docs.scvi-tools.org/en/stable/tutorials/notebooks/scrna/MrVI_tutorial.html. The source code and scripts used in this study (including implementations of the competing methods) are available at our GitHub repository: https://github.com/Haoran-Liu/SCDRL.
